# Collinsonin

**Published:** 1911-02

**Authors:** H. H. Redfield

**Affiliations:** Professor of Therapeutics, Illinois Medical College; Department of Medicine, Boyola University, Chicago, Illinois; Professor of Therapeutics and Physiology, Reliance Medical College, Chicago, Illinois


For Texas Medical Journal.

### Collinsonin.

BY H. H. REDFIELD, M. D., PROFESSOR OF THERAPEUTICS, ILLINOIS MEDICAL COLLEGE; DEPARTMENT OF MEDICINE, BOYOLA UNIVERSITY, CHICAGO, ILLINOIS; PROFESSOR OF THERAPEUTICS AND PHYSIOLOGY, RELIANCE MEDICAL COLLEGE, CHICAGO, ILLINOIS.

Collinsonin is a concentration prepared from Collinsonia Canadensis, common name Stone Root, a plant belonging to the natural order of Labiatae, and indigenous to the United States. Dose of the standard granule, gr. one-sixth.

*Physiological Action.*—Collinsonin has a stimulating effect upon the stomach, possesses powers as a diuretic, and produces a congestion of the venous radicles most pronounced in the pelvic organs.

*Therapeutics.*—Collinsonin is most frequently indicated in cases which present with hemorrhoids and constipation, and in which the patient speaks of a sensation of fullness in the rectum as though a foreign substance had found lodgment there. In these cases there is present a marked deficiency in the venous circulation.

The hemorrhoids and surrounding membranes are of a dark purplish, grape color, always associated with constipation, and are of recent origin.

While collinsonin is serviceable in all cases of hemorrhoids of recent origin, when there is a coexistent constipation or flatulence, its especial field seems to be in the treatment of those cases of hemorrhoids which appear during the later months of pregnancy or as a sequela of pregnancy.

The piles in these cases are very painful and often bleed. The constipation may alternate with diarrhea, or there may be dysentery with great tenesmus and a sensation as though the rectum were packed solid.

During the last months of pregnancy it is that the conditions just described are most often met with, and it is with these women that collinsonin is of the greatest benefit.

In some cases of pregnancy there may be an attendant pruritus which is sufficiently marked to keep the patient in a continual state of unrest. Whenever these conditions occur and the rectal symptoms and constipation which are so typical of collinsonin are present, the exhibition of this remedy will give excellent results.

Any conditions showing a depletion or enfeebling of the venous circulation, such as catarrhal gastritis, chronic pharyngitis and bronchitis, when the membranes are of the typical grape color, and the parts relaxed, collinsonin should be given consideration.

Properly understood and exhibited whenever the symptoms call for it, collinsonin is a very valuable remedy, and one that should be used oftener than it has been.

Compare its action with that of aesculin, hamemelin, dioscorein and baptisin.

